# Intelligence

**Published:** 1809-11

**Authors:** 


### INTELLIGENCE.

On Wednesday, the 18th ult. being the Festival of St. Luke, the College of Physicians held their Anniversary Meeting for the Harveyan Oration. Great expectations were entertained from the well-known acquirements of the Orator, (Dr. Heberden) who claims a sort of hereditary character as a scholar, a member of St. John's, Cambridge, and a Fellow of the London College. These expectations were not disappointed.

The Oration was throughout classical and perspicuous, delivered in a deliberate manner, and in a deep tone of voice, easily heard throughout the theatre, which was well attended. After mentioning most of the more celebrated characters who have adorned the College, the name of the late Dr. Heberden, the Orator's father, occurred in its order, and produced a pleasing melancholy effect on the whole audience. The preceding year furnished but too much scope for eulogy, by the death of three distinguished members, Sir George Baker, Dr. John Hunter, and Dr. Pitcairn. The two former having been authors, it was easy to commemorate by their writings as well as their characters. Dr. Pitcairn having left nothing but an exalted character, by which he could have been marked, furnished furnished fewer materials for panegyric. But the general feelings required none to produce a general sympathy, which was most delicately improved by the apostrophe with which his name was introduced. The figure was truly classical, the address being to the second person.

On the whole, we never witnessed an oration that was heard with more attention, or that gave more universal satisfaction. We trust it will be printed.

As it is not the courtesy to introduce the names of recently defunct licentiates, no mention was of course made of Sir J. Macnamara Hayes. This was the only one of that body who died last year. Among the licentiates in Midwifery, we have to lament Dr. Poignand; and among the extras, Dr. Manning.

The new names added to the list of candidates for Fellowships are,

Dr. James Cholmeley.
Dr. T. C. Morgan.
Dr. Richard Simmons.

To the list of Licentiates, no less than 14.

| | |
|---|---|
| Dr. A. Henderson. | Dr. R. B. Tomkins. |
| Dr. H. Edgworth. | Dr. Joseph Adams. |
| Dr. C. Whittell. | Dr. Alexander Lawler. |
| Dr. E. G. Jones. | Dr. J. Macleod. |
| Dr. J. De C. Laffan. | Dr. Thomas Hancock. |
| Dr. Mark Roget. | Dr. J. Penderleath. |
| Dr. Edmond Lockyer. | Dr. John Booth. |

Extra Licentiates.

Dr. J. Gasking | Dr. R. Anderson.
Dr. J. F. Berger.

The President continues in his office; and the Censors for the succeeding year are, Drs. Hervey, Morris, Willis, and Nevinson.

---

M. Alexandre, of Bourdeaux, employs a simple method of filtering water, without either sand, sponge, or pounded charcoal. It consists in merely causing the liquid to pass through the capillary tubes of a piece of half-worn out cotton. It is well known that a skein of thread, or a ribbon, one end of which is put into a vessel, while the other hangs over the side, will very soon become a conductor of the liquid, which filters and runs off, till the vessel is nearly empty. This experiment, M. Alexandre has applied on a large scale, to the purification of the water of the Garonne.

The acid, denominated pyrolignite of iron, obtained by the distillation of wood, is employed with great success in the arts. It has already been extracted from vinegar, without any empyreumatic smell, also from oil; and with it may be formed the base of a great number of solvents. M. Vitalis has applied it to the dying ing of thread and cotton, and this practice is now followed in the manufactories of Rouen, where black cottons for mourning, which used formerly to be procured from Holland, are dyed in a solid and cheap manner, by means of the pyrolignite of iron. This colour lasts very long, and is not liable to turn rusty like common blacks.

The following method of preserving grapes is given in a French Journal. Take a cask or barrel inaccessible to the external air, and put it into a layer of bran dried in an oven, or of ashes well dried and sifted. Upon this, place a layer of bunches of grapes well cleaned, and gathered in the afternoon of a dry day, before they are perfectly ripe. Proceed thus with alternate layers of bran and grapes, till the barrel is full, taking care that the grapes do not touch each other, and to let the last layer be of bran; then close the barrel, so that the air may not be able to penetrate, which is an essential point. Grapes thus packed will keep nine or even twelve months. To restore them to their freshness, cut the end of the stalk of each bunch of grapes, and put that of white grapes into white wine, and that of the black grapes into red wine, as you would put flowers into water, to revive or to keep them fresh.

Early in next month, Dr. Buxton will publish an Essay on the Use of a regulated Temperature in Winter Cough and Consumption; including Observations on the different methods of producing such a temperature in the chambers of invalids.

